# SGLT2 inhibitors improve kidney function and morphology by regulating renal metabolic reprogramming in mice with diabetic kidney disease

**DOI:** 10.1186/s12967-022-03629-8

**Published:** 2022-09-14

**Authors:** Yong-Ping Lu, Ze-Yu Zhang, Hong-Wei Wu, Li-Jing Fang, Bo Hu, Chun Tang, Yi-Qing Zhang, Lianghong Yin, Dong-E. Tang, Zhi-Hua Zheng, Ting Zhu, Yong Dai

**Affiliations:** 1 Department of Nephrology, Center of Kidney and Urology, The Seventh Affiliated Hospital, Sun Yat-Sen University, Shenzhen, China; 2 Department of Nephrology, The First Affiliated Hospital of Jinan University, Guangzhou, China; 3 Guangdong Provincial Engineering Research Center of Autoimmune Disease Precision Medicine, The Second Clinical Medical College of Jinan University, The First Affiliated Hospital of Southern University of Science and Technology, Shenzhen People’s Hospital, Shenzhen, 518020 China; 4 Department of Nephrology, Guangzhou First People’s Hospital, Guangzhou, China

## Abstract

Diabetic kidney disease (DKD) is the leading cause of end-stage renal disease (ESRD) worldwide. SGLT2 inhibitors are clinically effective in halting DKD progression. However, the underlying mechanisms remain unclear. The serum and kidneys of mice with DKD were analyzed using liquid chromatography with tandem mass spectrometry (LC–MS/MS)-based metabolomic and proteomic analyses. Three groups were established: placebo-treated littermate db/m mice, placebo-treated db/db mice and EMPA-treated db/db mice. Empagliflozin (EMPA) and placebo (10 mg/kg/d) were administered for 12 weeks. EMPA treatment decreased Cys-C and urinary albumin excretion compared with placebo by 78.60% and 57.12%, respectively (p < 0.001 in all cases). Renal glomerular area, interstitial fibrosis and glomerulosclerosis were decreased by 16.47%, 68.50% and 62.82%, respectively (p < 0.05 in all cases). Multi-omic analysis revealed that EMPA treatment altered the protein and metabolic profiles in the db/db group, including 32 renal proteins, 51 serum proteins, 94 renal metabolites and 37 serum metabolites. Five EMPA-related metabolic pathways were identified by integrating proteomic and metabolomic analyses, which are involved in renal purine metabolism; pyrimidine metabolism; tryptophan metabolism; nicotinate and nicotinamide metabolism, and glycine, serine and threonine metabolism in serum. In conclusion, this study demonstrated metabolic reprogramming in mice with DKD. EMPA treatment improved kidney function and morphology by regulating metabolic reprogramming, including regulation of renal reductive stress, alleviation of mitochondrial dysfunction and reduction in renal oxidative stress reaction.

**Supplementary Information:**

The online version contains supplementary material available at 10.1186/s12967-022-03629-8.

## Introduction

Diabetes mellitus (DM) is a common chronic metabolic disease characterized by persistent hyperglycemia caused by absolute or relative insulin deficiency and insulin resistance. The estimated number of patients with DM is projected to increase to 700 million by 2045 worldwide [1]. In addition, DM is the leading cause of chronic kidney disease (CKD) and end-stage renal disease (ESRD). Approximately 40% of patients with DM gradually progress to CKD and even develop ESRD [2]. Unfortunately, the treatment of DKD remains an unresolved challenge worldwide. To date, strict glycaemic and blood pressure control, especially with the use of angiotensin-converting enzyme inhibitors and angiotensin II receptor blockers, has been shown to delay the progression of DKD [3, 4]. However, the benefit of these interventions to DKD outcomes is limited [5]. Therefore, developing new treatment strategies to delay the progression of DKD is necessary.

The pathogenesis of DKD is multifactorial and still unclear. Accumulating evidence elucidates that various cellular stress responses triggered by metabolic abnormalities contribute to the pathophysiological mechanism of DKD [6]. The upregulation of sodium-glucose cotransporter 2 (SGLT2), when appearing in diabetes, leads to high transmembrane transport in the proximal tubule and generates a dramatic energy requirement in the renal cortex [7]. Meanwhile, metabolic reprogramming characterized by tricarboxylic acid cycle (TCA cycle) inhibition and glycolysis enhancement occurs in the renal cortex [8]. This phenomenon of mitochondrial oxidative phosphorylation activity mismatched with increased glycolytic flux results in the accumulation of intermediates from glucose metabolism and triggers multiple pathogenic signaling pathways, thereby inducing oxidative stress, reductive stress, and various cellular stress responses mediated by energy insufficiency [9, 10]. Moreover, insulin resistance shifts the pattern of renal biofuel utilization from glucose-mediated aerobic metabolism to fatty acid β-oxidation and amino acid metabolism [7]. Reversing these metabolic abnormalities has demonstrated encouraging results in improving the outcomes of DKD. For example, inhibited KIM-1-mediated fatty acid uptake in renal tubular epithelial cells can block a series of kidney injury-related events triggered by abnormal lipid metabolism in DKD [11]; similarly, the reversal of abnormal glycolysis and lipid metabolism in DKD was protective against the pathophysiology of DKD [8].

SGLT2 inhibitors originally developed for treating type 2 Diabetes mellitus (T2DM) are clinically very effective in halting the progression of DKD [12–14]. Recent studies have demonstrated that SGLT2 inhibitors exert both direct or indirect protective effects on the cardiovascular and renal systems in T2DM, which are independent of their glucose control effects but related to their effects on blood pressure control, glomerular hemodynamic amelioration, RAAS regulation, and anti-inflammation and in reducing glucotoxicity, lipotoxicity, and uric acid. Furthermore, SGLT2 inhibitors downregulate hepcidin and promote erythropoiesis [15, 16], thereby alleviating renal hypoxia by improving the circulating oxygen supply [7]. Interestingly, a recent study has demonstrated that dapagliflozin prevents the high glucose-induced metabolic transition shift from lipid oxidation to glycolysis in renal tubular cells by inhibiting HIF-1α [17]. In contrast, empagliflozin inhibit the over-activated nutrient-sensing signaling pathway by increasing endogenous ketone body production, thus improving energy utilization and reducing renal oxidative stress [18]. This suggests that the SGLT2 inhibitor effectively corrects metabolic disorders in DKD. However, the mechanisms behind the renoprotective effect of SGLT2 inhibitors are unclear.

This study was aimed to investigate the metabolic effects of SGLT2 inhibitors in DKD via liquid chromatography with tandem mass spectrometry (LC–MS/MS)-based metabolomic and proteomic analyzes of serum and the kidneys, and to provide a deeper understanding of the renoprotective mechanism of SGLT2 inhibitors.

## Research design and methods

### Diabetic kidney disease model

**Fig. 1:**
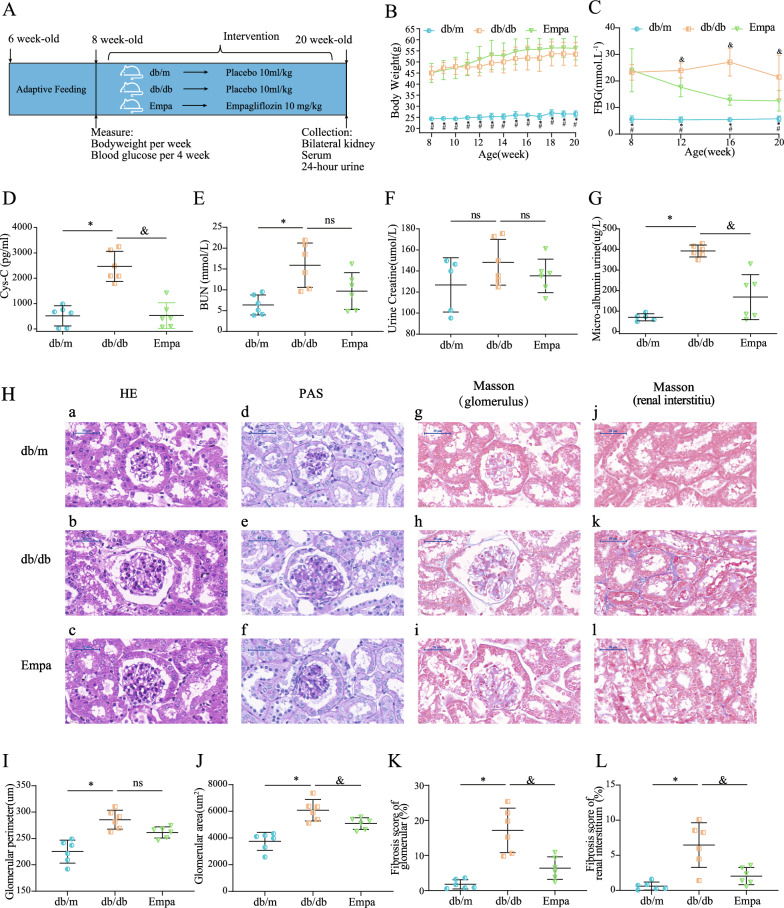
Effect of empagliflozin on the biochemical assay and renal pathology of db/db mice. **A** The workflow of the animal experiment. **B** Bodyweight changes of mice during the intervention. **C** Fasting-blood glucose levels of mice during the intervention. **D**–**G** The level of serum cystatin C (**D**), blood urea nitrogen (**E**), urine creatinine (**F**), urine microalbumin (**G**) in different groups. **H** Representative renal tissue pathology (× 200) of mice in each group (H&E staining, PAS staining, and Masson staining). **I**, **J** The average glomerular perimeters (**I**) and glomerular area (**J**) based on 10 randomly consecutive fields in each section from H&E staining. **K**, **L** The percentages of glomerular (**K**) and interstitial (**L**) fibrosis based on 10 randomly consecutive fields in each section from Masson staining. “*”, “#”, and “&” mean *p* < 0.05 when db/m vs. db/db, Empa vs. db/m, and Empa vs. db/db, respectively. “ns”, not significant

Six-week-old male db/db mice (C57BLKS/J-leprdb/leprdb, n = 30) and matched littermate db/m mice (C57BLKS/J-leprdb/leprm, n = 15, as normal controls) were purchased from the Nanjing University Experimental Animal Center. After 2 weeks of adaptive feeding, db/db mice were randomly divided into the db/db (model) and empagliflozin (EMPA; EMPA-treated) groups (n = 15). Mice in the EMPA group were administered 10-mg/kg EMPA per day via oral gavage for 12 weeks. Similarly, mice in the model and control groups were administered 10-mL/kg sterile water was dosed to the mice in the model and control group in the same way. All mice were housed in a controlled SPF-grade environment (temperature, 22 ± 2 ℃; humidity, 55 ± 10%; with a 12-h light/dark cycle) without dietary and drinking water restriction. At 3 weeks and every 4 weeks thereafter, whole blood was collected from the tail veins of mice after overnight fasting, and their fasting blood glucose levels were measured using a portable glucometer. All mice were weighed once a week beginning at the third week. In addition, 24-h urine samples were obtained from each mouse using metabolic cages after 12 weeks of treatment and stored at − 80 ℃ after they were snap-frozen in liquid nitrogen. Subsequently, the mice were anesthetized using 2,2,2-tribromoethanol via intraperitoneal injection, and whole blood samples were obtained via cardiac puncture. The samples were incubated at room temperature for 1 h in procoagulant tubes and centrifugated at 3000 r/min for 15 min to collect the upper serum. For kidney specimens, one side of the kidney was snap-frozen in liquid nitrogen after weighting, whereas the other side was fixed in 4% paraformaldehyde overnight and subsequently embedded in paraffin. All samples were stored at − 80 ℃ before performing LC–MS/MS. All experiments in this study were conducted according to the requirements of the National Law for Laboratory Animal Experimentation and were approved by the Commission on Experimental Animal Ethics of Jinan University (No. 202069-04). A detailed flowchart of animal experiments is shown in Fig. 1A.

### Biochemical analysis and histopathological examination

The fasting blood glucose level was detected using a portable glucometer (BAYER, Germany) with blood glucose test strips (BAYER, Germany). The serum and urine samples were thawed at 4 ℃ before detection. Serum cystosin C (Cys-C) levels were measured with a mouse Cys-C ELISA kit (E-EL-M3024, Elabscience, Wuhan, China). Urine creatinine (UCR) and urine microalbuminuria albumin levels were measured using a UCR enzyme-linked immunoassay kit (MM-44289M1, Elabscience, Wuhan, China) and MAU/ALB enzyme-linked immunoassay kit (MM-0705M1, Elabscience, Wuhan, China), respectively.

For renal histopathological analysis, paraffin-embedded renal tissues were cut into 4-um sections and subjected to haematoxylin–eosin (H&E), Masson and PAS staining. Subsequently, 10 randomly selected non-overlapping areas (25 × 25 μm) on HE-stained images (200×) were used to evaluate the extent of glomerular damage by calculating the average glomerular perimeter and area using the ImageJ software. The same method was used to select 10 fields of view on Masson-stained images and determine the percentage of the fibrotic area (blue-stained area) in each observed field for assessing the degree of glomerular and interstitial fibrosis. Renal histopathological analysis was performed by two independent investigators using a blinded method.

### Proteomic analysis

#### Sample preparation

Proteomic analysis was conducted as reported previously [19]. Briefly, an appropriate amount of renal tissue solubilised with 300 uL of RIPA lysis buffer was homogenised before sonication. Thereafter, the homogenates were centrifuged at 4 ℃ and 12,000 rpm for 10 min to collect the supernatant. The supernatant was precipitated using 80% pre-cold acetone and washed thrice, and the precipitates were redissolved in protein resolve buffer (HEPES­SDC1). Furthermore, serum samples were incubated in a water bath and used to extract proteins using the BIO-RAD de-high abundance protein kit. Thereafter, we determined protein concentration using a BCA kit. Subsequently, 100-ug total protein from each sample was diluted 5 times with 50-mM NH4HCO3 and reduced and alkylated with DTT (5 mM) and IAA (10 mM), respectively. Thereafter, the sample was mixed with 0.5-ug/uL trypsin (v:v, 50:1) and incubated overnight at 37 ℃ to obtain peptide solution for further LC–MS/MS analysis, and the reaction was quenched by adding 1-uL TFA.

#### LC–MS/MS for proteomic analysis

We used a nano-UPLC system (EASY­nLC1200) in combination with a mass spectrometer (Q­Exactive HFX) equipped with a nano-litre ion source for proteomic data acquisition. A 100-um ID × 15 cm reversed-phase column (ReprosilPur 120 C18AQ, 1.9 um, Dr. Math) was used for chromatographic separation. Mobile phase A contained 0.1% formic acid aqueous solution with 2% acetonitrile, whereas mobile phase B contained 0.1% formic acid aqueous solution with 80% acetonitrile, and the flow rate was 300 nL/min. MS was performed based on data-dependent acquisition (DDA) in the positive ion mode. The parameters were set as follows: full scan range, 350–1600 m/z; resolution, 120 K; AGC target, 3E6; maximum ion implantation time, 50 ms. The top 20 highest-intensity ions were fragmented via HCD with the normalised crash energy (NCE) of 27%, and the quadrupole isolation window was 1.2 m/z. The dynamic exclusion time was set to 45 s, and single-charged peaks and peaks with charges > 6 were excluded from DDA.

#### Data processing

Subsequently, raw MS data were processed using the Proteome Discoverer (PD) software (Version 2.4.0.305) and the plug-in Sequest HT search engine. The MS data were matched according to the species-level UniProt FASTA databases (uniport-Mus + musculus-10090-2020-10.fasta). Carbaminomethyl (C) was considered a fixed modification, whereas oxidation (M) and acetyl (protein N term) were considered variable modifications. Trypsin was used as a protease. Peptide tolerance was set to 10 ppm, and MS/MS tolerance was set to 0.02 Da. Only two missed cleavages were acceptable. The PSM and peptide levels were set as the false discovery rate (FDR) of 0.01. The quantification of proteins and normalisation of the total peptide amount were performed based on the unique and razor peptides. Other parameters were maintained as default. The detailed process of metabolomic analysis is described below.

### Metabolomic analysis

#### Metabolite extraction

For metabolite extraction, 25 mg of each kidney sample was taken and mixed with 500 uL of the extract solution (methanol:acetonitrile:water = 2:2:1, with isotopically-labelled internal standard mixture). The samples were homogenised at 35 Hz for 4 min and subsequently sonicated in an ice-water bath for 5 min. These steps were repeated twice or thrice. The serum samples were thawed at room temperature, and 50 uL of each sample was mixed with 200 μL of the extract solution in an EP tube, vortexed for 30 s and sonicated in an ice-water bath for 5 min. Thereafter, the processed kidney and serum samples were incubated at − 40 ℃ for 1 h and centrifuged at 12,000 rpm for 15 min at 4 ℃. The supernatant was collected in a sample bottle for further detection. In addition, an equal volume of all samples was stored for subsequent quality control (QC) of LC–MS/MS analysis.

#### LC–MS/MS analysis

A UHPLC system (Vanquish, Thermo Fisher Scientific) with UPLC BEH Amide (2.1 mm × 100 mm, 1.7 μm) combined with a Q Exactive HFX mass spectrometer (Orbitrap MS, Thermo) was used for LC–MS/MS analysis. Mobile phase A comprised 25-mmol/L ammonium acetate and 25 ammonia hydroxide in water (pH = 9.75); mobile phase B was acetonitrile. The temperature of the auto-sampler was maintained at 4 ℃, and the injection volume was 3 μL. All data, including MS and MS/MS data, were obtained using the QE HFX mass spectrometer under the control of an acquisition software (Xcalibu, Thermo). The detailed parameters of ESI source conditions were as follows: sheath gas flow rate, 30 Arb; aux gas flow rate, 25 Arb; capillary temperature, 350 ℃, full MS resolution, 60,000; MS/MS resolution, 7500; collision energy, 10/30/60 in the NCE mode; spray voltage, 3.6 kV (positive) or − 3.2 kV (negative).

#### Data processing

The ProteoWizard software was used to convert the format of the raw data to mzXML. The converted data were processed using an in-house R package based on XCMS for peak detection, extraction, alignment and integration. Subsequently, metabolites were annotated by matching with a secondary MS database based on the self-built BiotreeDB (V2.1) with the cutoff for annotation set to 0.3. Missing values in raw data were filled using half of the minimum value, and all data were normalised using the total ion intensity (TIC) of every single sample for the convenience of further analysis.

### Normalization and imputation of proteomic and metabolomic analyses

For the raw proteomic and metabolomic data, the relative intensity of each protein was normalised by log2 conversion, whereas the relative intensity of each metabolite was normalised based on the total ion current (TIC) of each metabolite. A protein or metabolite was discarded if it was detected in < 50% samples. Otherwise, the protein or metabolite was retained, and the missing value was replaced with half of the minimum positive value of each variable.

### Bioinformatic analyses

Multivariate statistical analysis was performed, in which unsupervised principal component analysis (PCA) was conducted to observe the metabolic profile of all subjects, identify abnormal outliers and assess the stability of QC samples. In addition, supervised partial least square-based discriminant analysis (PLS-DA) was used for characterising metabolic feature separation among different groups and screening biomarkers. Q2 and R2 were the parameters for evaluating the predictability and interpretability and testing the fit of the PLS-DA model via leave-one-out cross-validation. The variable importance in projection (VIP) score was calculated based on PLS-DA to quantify the contribution of metabolites in the model and was used to screen for differentially expressed metabolites. Metabolites with VIP scores > 1, P-value < 0.05 (Student’s t‐test) and |fold change|> 1.5 were considered significantly differentially expressed metabolites (DEMs). Similarly, proteins with |fold change|> 1.5 and adjusted P-value < 0.05 were considered differentially expressed proteins (DEPs). The Short Time-series Expression Miner (STEM) program was used to identify target proteins regulated by EMPA. A Venn diagram, volcano plots and a hierarchically clustered heat map were generated using the R packages ‘ggplot’ and ‘Complexheatmap’ to visualise the feature of identified DEPs and DEMs. Gene Ontology (GO) enrichment analysis and KEGG enrichment analysis were used to identify the potential pathways of differentially expressed proteins and metabolites and EMPA-altered proteins and metabolites. To quantify the overall expression of each enriched metabolic pathway, the average intensity of all metabolites in a pathway with at least three metabolites was assessed and used to calculate the fold change of each pathway, which indicated the activity of the pathway [20]. The identified metabolites were classified according to the HMDB database, and the percentage of each metabolite category was calculated. Thereafter, the Fisher’s exact test or chi-square test was used to evaluate changes in the percentage of each metabolic category before and after EMPA intervention.

### Statistical analyses

Biochemical indicators were analyzed using SPSS Statistics version 25.0 (IBM, USA), GraphPad Prism 9 and R software (R Foundation for Statistical Computing, Vienna, Austria, v3.5.3). The Student’s t-test was used for comparing data between two groups. Normally distributed data were expressed as mean ± SD. Comparison among multiple groups and pairwise comparison were performed using one‐way ANOVA and LSD test, respectively. The relationship between the serum and kidney in both metabolomic and proteomic analyses was assessed using Pearson correlation analysis. A P-value of < 0.05 was considered statistically significant.

## Results

### Effects of EMPA on biochemical indicators and pathological characteristics in db/db mice

The flowchart of the animal experiment is shown in Fig. 1A. Mice in the db/db and EMPA groups had higher body weight than mice in the db/m group, and no difference was observed in body weight between the db/db and EMPA groups (Fig. 1B). EMPA treatment improved fasting blood sugar (FBG), blood urea nitrogen (BUN), Cys-C and serum creatinine levels and urine albumin-to-creatinine ratio (P < 0.05) (Fig. 1C–G).

Subsequently, renal histopathological examination (Fig. 1H) revealed that db/db mice had conspicuous pathological alterations, such as glomerular dilatation, mesangial cell hypercellularity, mesangial matrix hyperplasia and tubular basement membrane thickening, which were corrected after EMPA treatment (Fig. 1H, a–f). As shown in Fig. 1I and J, EMPA treatment normalized the average glomerular perimeters (P = 0.070) and glomerular area (P = 0.045) in db/db mice, suggesting that EMPA mitigates glomerular hypertrophy in DKD. In addition, Masson staining showed a significant increase in renal fibrosis in db/db mice (Fig. 1H, g–l). EMPA intervention reduced the level of glomerular and interstitial fibrosis by 62.8% and 68.5%, respectively (Fig. 1K, L). These results indicate that EMPA can reduce urinary protein excretion, improve renal function and reverse the renal pathological alterations resulting from DKD to some extent, which was consistent with a previous study [21].

### Effects of EMPA on the protein expression profile of db/db mice

A total of 6009 renal proteins were detected in 6 db/m, 6 db/db and 5 EMPA samples using the DIA-based non-labeled quantitative method (Additional file 1: Fig. S1A, Additional file 2: Table S1). Of the 6009 renal proteins, 5010 (83.4%) were supported by at least 2 peptides, with an average number of 10 peptides (Additional file 1: Fig. S1B). In addition, 84% of proteins (5046) were detected in 17 kidney samples (Additional file 1: Fig. S1C). Similarly, 1481 serum proteins were detected in 6 db/m, 5 db/db and 6 EMPA samples (Additional file 1: Fig. S1D, Additional file 2: Table S2); of which, 74% of proteins were supported by > 2 peptides, and 762 proteins were co-expressed in 17 serum samples (Additional file 1: Fig. S1E). Therefore, the quantification results of the proteomic and metabolomic analyses of the kidney and serum are highly credible.

**Fig. 2:**
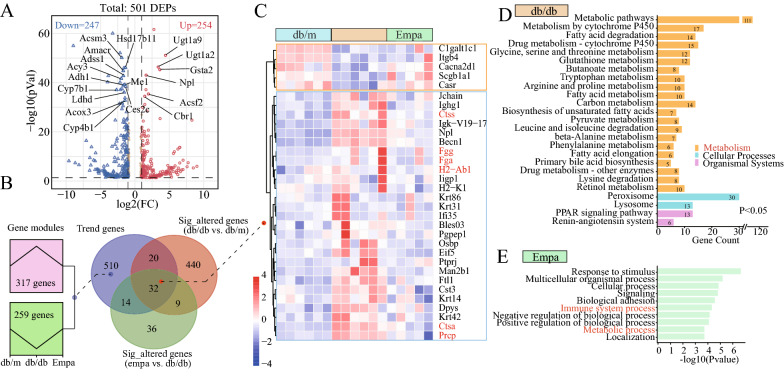
Effect of empagliflozin on the protein expression profile in the kidney of db/db mice. **A** Volcano plot showing the 501 differentially expressed proteins in the kidney of db/db mice, compared with the db/m group (all points meet |fold change|> 1.5, adjusted p < 0.05; among them, red and blue points have |fold change|> 2). Metabolism-related proteins were highlighted in the plot. **B** Left, Module plot depicting gene changes among groups (Minimum fold change for gene screening = 1.5). The red module shows 317 trend proteins up-regulated in the db/db group and down-regulated after empagliflozin therapy. The green module shows 259 trend proteins down-regulated in the db/db group and up-regulated after empagliflozin therapy; Right, venn plot showing the intersection of significantly changed proteins (|fold change|> 1.5, and adjusted *p* < 0.05) in the db/db and Empa groups and the above trend proteins. **C** Heatmap depicting the expression levels of the 32 potential target proteins regulated by empagliflozin. Key proteins that were reported to be associated with diabetes or/and diabetic nephropathy were marked with red. **D** KEGG pathway identification of the differentially expressed proteins in the kidney of db/db mice. **E** Go enrichment of changed proteins in the Empa group

In renal proteomic analysis, 501 DEPs were identified in the db/db group (Additional file 2: Table S3), and metabolism-related proteins (e.g. ugt1a9, ugt1a2, gsta2, acsf2, npl, acsf2, cbr1, acsm3, amacr and acy3) dominated the top altered proteins, revealing dramatic changes in metabolic processes in the kidney of db/db mice (Fig. 2A). To identify target proteins regulated by EMPA, we first identified 91 DEPs in the Empa group (Additional file 2: Table S3), and 576 trend proteins that varied between groups (Fig. 2B); of which, 32 proteins were significantly altered in the kidneys of db/db mice and the effects were considerably reversed after EMPA treatment(Fig. 2B and C, Additional file 2: Table S4). Among these proteins, high levels of fibrinogen proteins (Fgg and Fga), cathepsin proteins (ctss and ctsa), H2-Ab1 and prcp were considered risk factors for diabetic nephropathy and the predictors of urinary protein progression [22–26]. Pathway and GO enrichment analyses showed that altered proteins in the db/db group were significantly enriched in metabolic pathways, the PPAR signaling pathway and the renin–angiotensin system (Fig. 2D). However, EMPA therapy remarkably altered the metabolic process and immune response in the kidney of db/db mice (Fig. 2E).

**Fig. 3:**
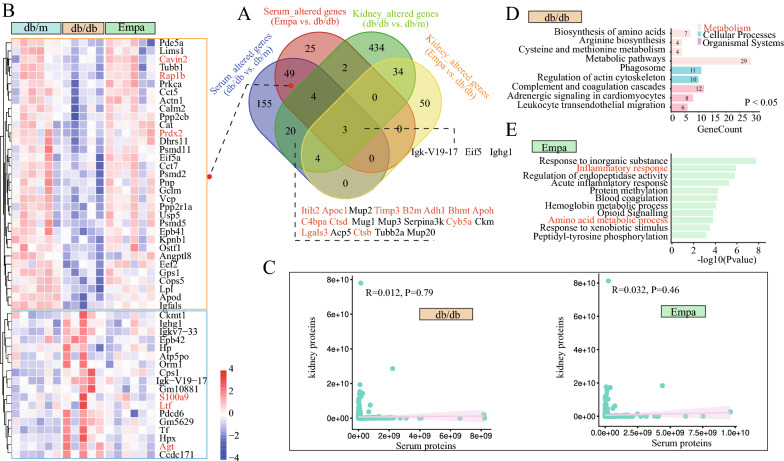
Effect of empagliflozin on the serum protein expression profile of db/db mice. **A** Venn plot showing the intersection of significantly changed proteins (fold change > 1.5 or < 0.86, and adjusted *p* < 0.05) in the serum and kidney samples. Proteins marked with red represented known diabetes-associated proteins. **B** Heatmap depicting the expression levels of the 51 potential target proteins regulated by empagliflozin. **C** Scatter plot showing the Pearson’s correlation between serum and kidney proteins. **D** KEGG pathway identification of the differentially expressed proteins in the serum of db/db mice. **E** Go enrichment of changed proteins in the Empa group

Simultaneously, we detected 1481 proteins in serum samples, including 235 and 83 DEPs in the db/db and EMPA groups, respectively (Fig. 3A, Additional file 2: Table S5). Among the intersection of altered serum proteins in the db/db and EMPA groups, 33 downregulated and 18 upregulated proteins were identified in the db/db group, whose expression was significantly reversed after EMPA therapy (Fig. 3B, Additional file 2: Table S6). Among these proteins, decreased levels of cavin2, rap1b and prdx2 were reported to be associated with insulin resistance [27], renal tubular mitochondrial dysfunction [28] and oxidative damage [29], respectively, whereas evaluated levels of Agt and s100a9 showed significant association with diabetic nephropathy [30] and diabetic retinopathy [31], respectively. In the db/db group, 20 proteins, including known diabetes-associated proteins such as Itih2, Apoc1, Timp3, B2M, Adh1, Bhmt, Apoh, C4bpa, Ctsd, Ctsb, Lgals3 and Cyb5a, were significantly altered in both serum and kidney samples, whereas only three identical proteins (Igk-V19-17, Eif5 and Ighg1) were detected in the EMPA group (Fig. 3A). This finding suggested that the drug effects of EMPA on the renal and circulating protein profiles were mutually independent. Furthermore, correlation analysis showed no significant correlation between circulating and renal tissue proteins in the db/db and EMPA groups (Fig. 3C). Pathway analysis revealed that altered serum proteins in the db/db group were significantly enriched in metabolic pathways, complement and coagulation cascades and leukocyte transendothelial migration (Fig. 3D). EMPA treatment substantially changed the amino acid metabolism and inflammatory response in the circulatory system of db/db mice (Fig. 3E). These results indicated that EMPA exhibited strong effects on metabolic processes in db/db mice.

### Metabolomic analysis

To systematically characterize the metabolic profile in DKD and the potential metabolic alterations after EMPA treatment, we performed non-targeted metabolomic analysis of the serum and kidney tissue in three experimental groups. We identified 897 (positive ion mode: 666 and negative ion mode: 231) and 678 (positive ion mode: 457, negative ion mode: 221) metabolites from the kidney and serum samples, respectively (Additional file 2: Tables S7 and S8).

In both positive and negative ion modes, extracted ion chromatograms (EIC) for the internal standard (IS) in quality control (QC) samples showed great consistency in retention time and response intensity (Additional file 1: Fig. S2A and B), indicating good stability for the acquisition of MS data in this study. In addition, Pearson correlation analysis revealed a high correlation among all QC samples (Additional file 1: Fig. S2C and D). Furthermore, three ISs were monitored, and their retention time and mass-to-charge ratio had satisfactory RSD (RSD median ≤ 15%) (Additional file 1: Fig. S2E and F). These results indicated that the sample quality, experimental method and system stability were reliable and suitable for metabolomic analysis of the kidney and serum.

#### Overall metabolic alterations in db/db mice

**Fig. 4:**
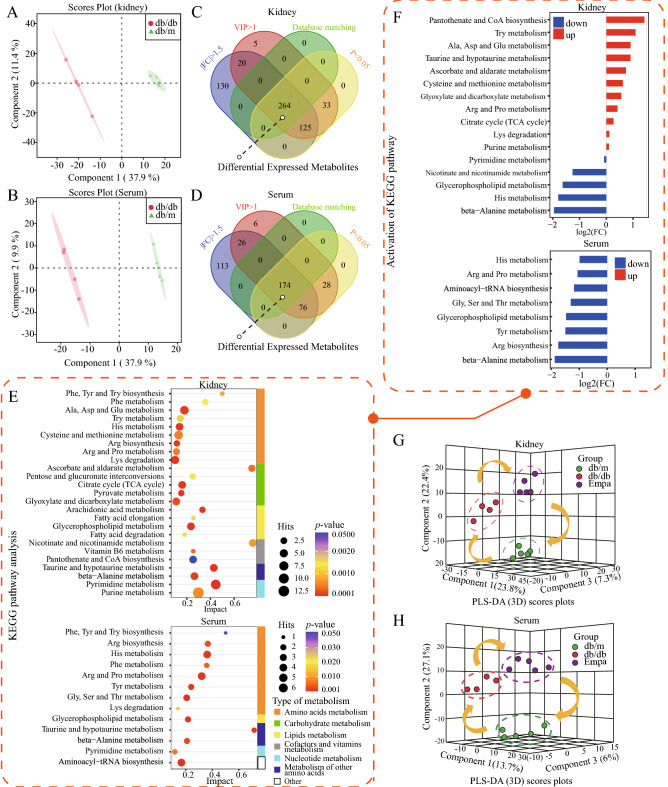
Disturbed metabolic profile in db/db mice and the entire metabolic alteration after the empagliflozin intervention. **A**, **B** PLS-DA score showing the conspicuous differential metabolic features of the kidney (**A**) and serum (**B**) between db/m and db/db group. **C**, **D** Venn plot showing the screening methods for 264 kidney DEMs (**C**) and 174 serum DEMs (**D**) between db/m and db/db group. Metabolites with |fold change|> 1.5, VIP > 1, *p* < 0.05, and matched in KEGG and HMDB database, are considered as differential expressed metabolites. **E** KEGG pathway analysis based on the relative intensity levels of 264 kidney DEMs and 174 serum DEMs identified between db/m and db/db groups (hypergeometric test, *p* < 0.05; Impact > 0.1). Those enriched pathways belong to 7 types of metabolism. **F** Activity state of enriched metabolic pathways in db/db mice. The average intensity of all metabolites in the pathway with at least three metabolites was used to calculate the fold change of each pathway to judge the activity state of the pathway. Red, elevated activity in db/db mice; Blue, decreased activity in db/db mice. **G**, **H** 3D PLS-DA scores plot summarising the overall metabolic alteration in the kidney (**G**) and serum (**H**) among db/m, db/db, and Empa groups

PLS-DA models showed noticeable differences in the distribution between db/db and db/m groups in the metabolomic analysis of both kidney (Q2 = 0.902, R2 = 0.999) and serum (R2 = 0.999, Q2 = 0.898) (Fig. 4A , B). According to the screening criteria of VIP > 1, P < 0.05 and |fold change|> 1.5, 389 and 174 DEMs were identified between the db/m and db/db groups in the kidney and serum, respectively. Of these DEMs, 264 renal and 174 serum metabolites were finally matched by comparing the KEGG and HDMB databases (Fig. 4C , D, Additional file 2: Tables S9 and S10).

To assess the aberrant alterations in metabolic pathways in DKD, we submitted 264 and 174 DEMs to Metaboanalyst 5.0 for KEGG pathway enrichment analysis. DEMs in the kidney were significantly enriched in 26 pathways (P < 0.05 and impact > 0.1; Fig. 4E, Additional file 2: Table S10), involving five types of metabolic abnormalities, including energy metabolism (glucose, lipid and amino acid metabolism), cofactor and vitamin metabolism, nucleotide metabolism and metabolism of other amino acids. The results indicated a reconfiguration of overall metabolism in db/db mice. Subsequently, we calculated the fold change of enriched pathways with at least three metabolites to assess their activity in the db/db and db/m groups (Fig. 4F). We found that the metabolic pathways of various amino acids, including Try, alanine, aspartic acid, glutamic acid, cysteine, methionine, arginine, proline, histidine, beta-alanine, glycine, serine, threonine and tyrosine metabolism; taurine and hypotaurine metabolism; lysine degradation and arginine biosynthesis, were altered in the kidney and serum, which highlighted the role of protein–energy wasting in DKD pathogenesis. In addition, nucleotide metabolism (e.g. purine and pyrimidine metabolism) and cofactor and vitamin metabolism (e.g. nicotinate and nicotinamide metabolism) were also altered in db/db mice.

Subsequently, we established a 3D PLS-DA model to examine the metabolic profiles of groups and observed a significant separation trend among them (Figs. 2H and 4G), which indicated that the construction of the DKD model was successful, and db/db mice had evident metabolic disorders. However, EMPA treatment reversed the changes resulting from DKD.

#### EMPA ameliorated kidney metabolism

**Fig. 5:**
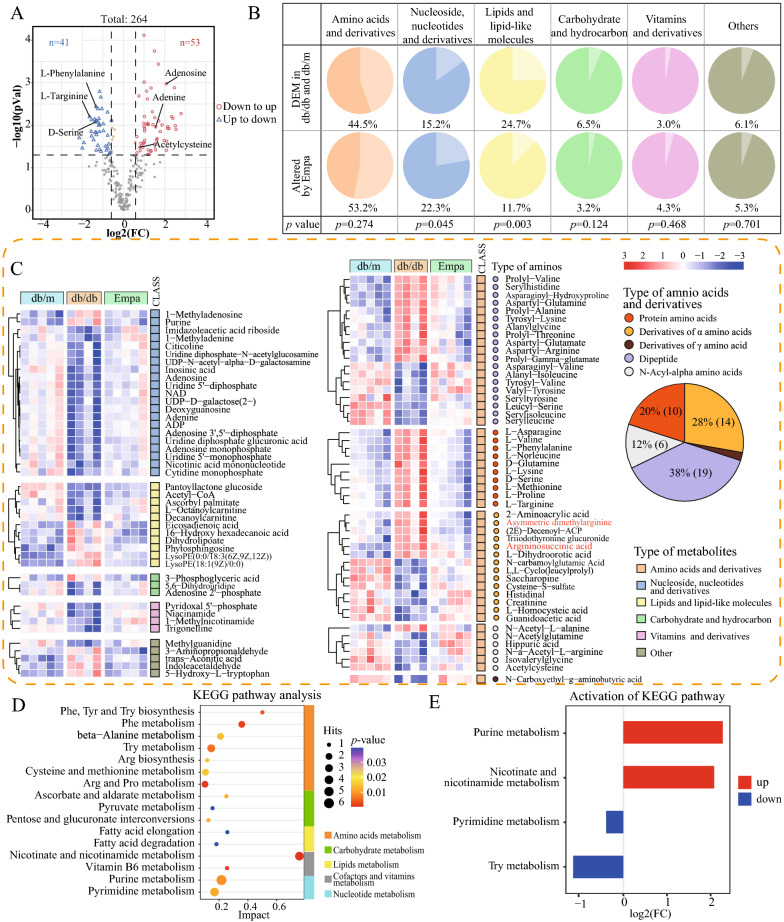
Empagliflozin ameliorated kidney metabolism. **A** Venn plot showing 94 Empa-altered metabolites in 264 kidney DEMs (|fold change|> 1.5, and *p* < 0.05). Renal damage-related metabolites were highlighted in the plot. **B** Pie charts showing the Chi-squared test (or Fisher exact test) of the percentage changes in the category of metabolites for the 264 DEMs in db/db mice and the 94 Empa-altered metabolites. **C** Heatmap depicting the expression levels of the 94 kidney metabolites altered by empagliflozin. Those 94 metabolites were classified into 6 categories according to the HMDB database. Among them, the metabolites belonging to ‘amnio acids and derivatives’ were further divided into five different types and calculated its percentage. Two known uremic toxins were highlighted. **D** KEGG pathway analysis based on the relative intensity levels of 94 Empa-altered metabolites in the kidney (hypergeometric test, *p* < 0.05; Impact > 0.1). **E** Activity state of enriched metabolic pathways for 94 Empa-altered metabolites in the kidney

We constructed a PLS-DA model to compare the metabolic characteristics between the db/db and EMPA groups in the kidney (Q2 = 0.54, R2 = 0.848**)** and identified 194 DEMs with the screening conditions mentioned earlier (Additional file 1: Fig. S3A and 3B, Additional file 2: Table S11). Of the 194 DEMs, 94 metabolites, co-expressed with 264 DEMs, were identified as EMPA-altered metabolites (EAMs) (Additional file 1: Fig. S3B); of which, the expression of 41 upregulated and 53 downregulated metabolites in the db/db group were significantly reversed after EMPA treatment (Fig. 5A, Additional file 2: Table S12). In addition, the expression of renal damage-related metabolites (e.g. l-phenylalanine, l-arginine and d-serine) was downregulated after EMPA treatment, whereas the expression of renal protection-related metabolites (e.g. adenosine, adenine and acetylcysteine) was increased in the kidney (Fig. 5A) [32–36].

Metabolism-related proteins (e.g. ugt1a9, ugt1a2, gsta2, acsf2, npl, acsf2, cbr1, acsm3, amacr and acy3) dominated the top altered proteins, revealing dramatic changes in metabolic processes in the kidney of db/db mice.

Subsequently, we classified 264 DEMs and 94 EAMs in the kidneys according to the HMDB database and used the chi-square test or Fisher’s exact test to estimate changes in the proportion of metabolite categories before and after EMPA treatment (Fig. 5B). We found that elevated proportion of the ‘nucleoside, nucleotides and derivatives’ (from 15.2% to 22.3%, P = 0.045) and reduced proportion of the ‘lipids and lipid-like molecules’ (from 24.7% to 11.7%, P = 0.003) categories were statistically significant.

Furthermore, hierarchical cluster analysis was performed on 94 renal EAMs (Fig. 5C). In the ‘nucleoside, nucleotides and derivatives’ category, most downregulated metabolites were upregulated after EMPA treatment, when compared to the db/db and db/m groups. KEGG enrichment analysis revealed that these nucleotide metabolites were significantly enriched in pathways related to purine and pyrimidine metabolism (Fig. 5D). In the ‘lipids and lipid-like molecules’ category, the expression of three fatty acids and three phospholipids (including two lysoPE) was elevated in db/db mice but decreased after EMPA treatment. In addition, the expression of two types of acylcarnitines and three fatty acyl classes (especially acetyl-CoA, which is the hub of energy metabolism in the body) was increased after EMPA intervention.

The ‘amino acids and derivatives’ category accounted for the largest proportion both before and after treatment. Among these amino metabolites, the proportion of dipeptides was the highest. The most noticeable change was observed in the expression of valyl-tyrosine (Val-Tyr, from downregulation to upregulation, FC [db/db versus db/m] = 0.24 and FC [EMPA versus db/db] = 6.94). In addition, there were 10 types of protein amino acids and 14 types of derivatives of α-amino acids in EMPA regulation. Five *N*-acyl-alpha amino acids, except for *N*-acetyl-l-alanine, were upregulated after EMPA treatment. It is noteworthy that *N*-acetylcysteine is a direct precursor to glutathione. We also observed that the expression of two known uremia toxins, argininosuccinic acid and impic dimethylarginine, was reduced after EMPA treatment (Additional file 2: Table S12).

We further performed KEGG pathway enrichment analysis on 94 EAMs in the kidney and assessed the activity of enriched pathways (Fig. 5D, E). The overall activity of purine metabolism (log2[FC] = 2.278, P = 0.009), nicotinate and nicotinamide metabolism (log2[FC] = 2.081, P = 0.0004), pyrimidine metabolism (log2[FC] = − 0.395, P = 0.0118) and tryptophan metabolism (log2[FC] = − 1.151, P = 0.006) was altered after EMPA treatment, as compared with the model group.

#### EMPA ameliorated serum metabolism

**Fig. 6:**
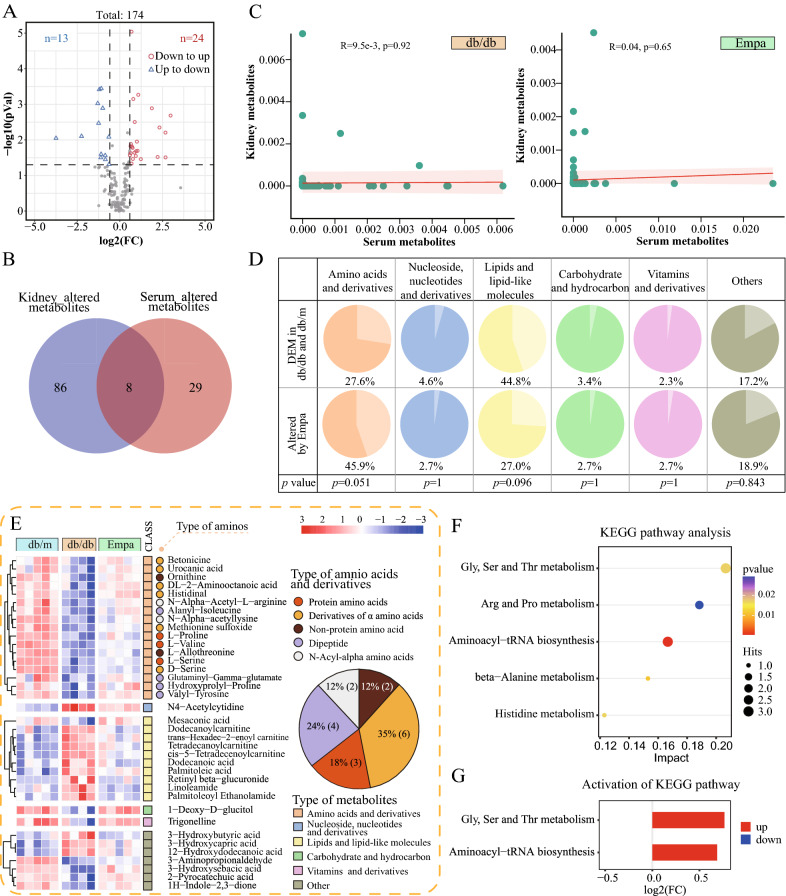
Empagliflozin ameliorated serum metabolism. **A** Venn plot showing 37 Empa-altered metabolites from 174 serum DEMs (|fold change|> 1.5, and *p* < 0.05). **B** The Venn plot shows the intersection of renal metabolites and serum metabolites altered by Empa. **C** Scatter plot showing the Pearson’s correlation between serum and kidney altered metabolites. **D** Pie charts showing the Chi-squared test (or Fisher exact test) of the percentage changes in the category of metabolites for the 194 serum’s DEMs in db/db mice and the 37 Empa-altered metabolites. **E** Heatmap depicting the expression levels of the 37 serum metabolites altered by empagliflozin. Those 37 metabolites were classified for 6 categories according to the HMDB database. Among them, the metabolites belonging to ‘amnio acids and derivatives’ were further divided into five different types and calculated its percentage. **F** KEGG pathway analysis based on the relative intensity levels of 37 Empa-altered metabolites in the serum (hypergeometric test, *p* < 0.05; Impact > 0.1). **G** Activity state of enriched metabolic pathways for 37 Empa-altered metabolites in the serum

We analyzed alterations in serum metabolism in db/db mice treated with EMPA. The results of PLS-DA in serum were consistent with metabolic alterations observed in the kidney (R2 = 0.994, Q2 = 0.483) (Additional file 1: Fig. S3C). A total of 174 serum metabolites were identified as DEMs between the db/db and EMPA groups using the same screening criteria mentioned earlier. Of the 174 metabolites, 37 (DEMs in the db/m and db/db groups) were identified as EAMs in serum (Additional file 1: Fig. S3D). As demonstrated by the volcano plot, the expression of 13 EAMs in serum was increased and that of 14 EAMs was decreased after EMPA treatment (Fig. 6A). In addition, only 8 EAMs were significantly altered in both serum and kidney samples (Fig. 6B). The correlation analysis revealed no significant relationship between serum and renal metabolites in the db/db and EMPA groups, suggesting the mutually independent effects of EMPA in renal and serum metabolic profiles (Fig. 6C).

Similarly, we classified 174 DEMs and 37 EAMs in serum according to the HMDB database and performed the Fisher’s exact test to distinguish the percentage change in metabolite classification. The results revealed that the proportion of the ‘amino acids and derivatives’ category was increased (from 27.6 to 45.9%, P = 0.051) after EMPA treatment (Fig. 6D) and was highest before and after treatment. In comparison, the proportion of other metabolite categories was not significantly different before and after treatment. A heat map demonstrating hierarchical clustering of 37 TRMs showed that the expression of all metabolites in the ‘amino acids and derivatives’ category was increased after EMPA treatment (Fig. 6E). The proportion of the ‘amino acids and derivatives’ category was ranked in order of ‘derivatives of α amino acids’ (35%, n = 6), ‘dipeptide’ (24%, n = 4), ‘protein amino acids’ (18%, n = 3), ‘*N*-acyl-alpha amino’ (12%, n = 2) and ‘non-protein amino (12%, n = 2)’.

Subsequently, KEGG pathway enrichment analysis was performed and the activity state of pathways associated with 37 serum EAMs were predicted. We observed that all significantly altered pathways in serum were related to amino metabolism, such as glycine, serine and threonine metabolism (P = 0.017, impact = 0.207); arginine and proline metabolism (P = 0.029, impact = 0.188); aminoacyl-tRNA biosynthesis (P = 0.0005, impact = 0.1667); beta-alanine metabolism (P = 0.011, impact = 0.153) and histidine metabolism (P = 0.017, impact = 0.123). In these significantly enriched pathways, the overall levels of metabolites in ‘glycine, serine and threonine metabolism’ (log2[FC] = 0.765) and ‘aminoacyl-tRNA biosynthesis’ (log2[FC] = 0.688) pathways showed a trend opposite to that of the db/db group after EMPA intervention (Figs. 4E and 6G).

#### Potential mechanisms of EMPA in protection against DKD

**Fig. 7:**
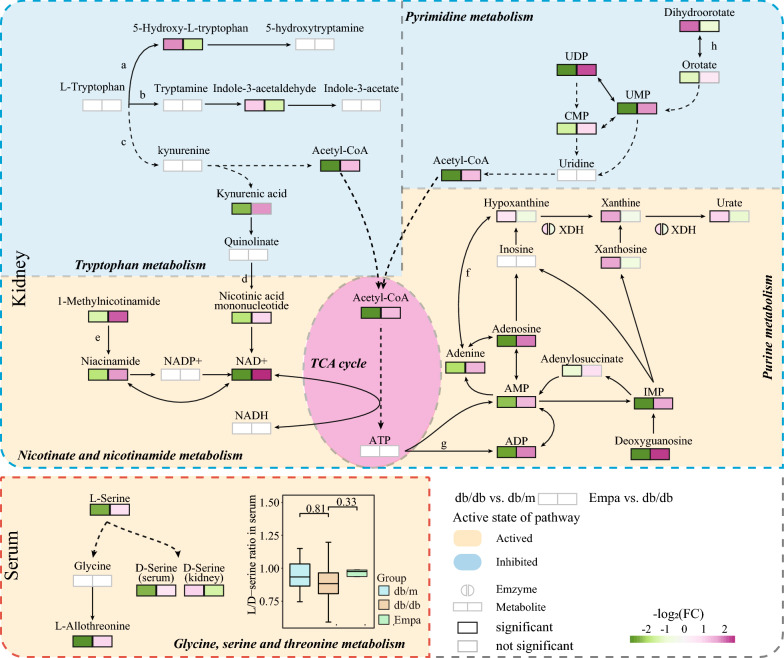
The metabolic network for the key pathways in the renoprotection effect of Empa in db/db mice. The background color for each pathway represents the active state after Empa intervention. Yellow, elevated activity after Empa intervention; Blue, decreased activity after Empa intervention. The color of the box below each metabolites represents the log2 (fold change) of this metabolite between db/db and db/m or Empa and db/db group. ‘a’, the serotonin metabolism pathway; ‘b’, the indole pathway; ‘c’, the kynurenine pathway; ‘d’, NAD+ de novo synthesis pathway; ‘e’, the salvage synthesis pathway of NAD+; ‘f’, purine degradation pathway; ‘g’, ATP-degradation mediated purine nucleotide conversion bypass; ‘h’, pyrimidine de novo synthesis pathway

As described above, we eventually identified four metabolic pathways associated with EMPA therapy in the kidney, including purine, nicotinate, nicotinamide, pyrimidine, tyrosine and β-alanine metabolism. These metabolism pathways are associated with the tricarboxylic acid (TCA) cycle through acetyl-CoA (Fig. 7). The ‘glycine, serine and threonine metabolism’ pathway was the most significantly altered pathway that responded to EMPA treatment in serum. It was related to the conformational transition between l-serine and d-serine. Altogether, we speculate that these metabolic pathways may play a critical role in the progression of DKD, and EMPA may exert beneficial effects by regulating these pathways.

## Discussion

In this work, we observed that Empa treatment effectively alleviates the renal pathological changes caused by T2DM; simultaneously, we characterized the specific feature of metabolic reprogramming that occurs in kidney and serum in the mice with DKD via multi-omics analysis and identified five metabolic pathways related to Empa treatment, which involve in renal purine metabolism (local purinergic signaling); pyrimidine metabolism (mitochondrial function); tryptophan metabolism (reductive stress); nicotinate and nicotinamide metabolism (reductive stress) and serum glycine, serine and threonine metabolism (nephrotoxicity). Our results suggest that EMPA treatment improves renal function and morphology by regulating renal metabolic reprogramming in mice with DKD.

In physiological conditions, the filtered amino acids are almost reabsorbed by tubular epithelial cells. Furthermore, the kidney is a crucial organ that participates in the biosynthesis and catabolism of multiple amino acids and their derivatives [37]. A previous study found that patients with DKD were more predisposed to develop amino acid metabolic derangements than patients with DM or healthy people [38]. In addition, a recent study confirmed that dapagliflozin intervention could reverse the abnormal expression of multiple amino acid transporters and amino acid degrading enzymes in the renal cortex of diabetic mice [39]. In this study, amino acids and their derivatives had the highest proportion of metabolites before and after EMPA treatment, whether in the serum or kidney. Among them, *N*-Acetylcysteine is the precursor to glutathione in vivo, which has a potent effect of scavenging oxygen free radicals and antioxidants by providing sulfhydryl to increase the synthesis of endogenous glutathione and thus reducing the oxidative damage caused by mitochondrial dysfunction [40]. A recent study confirmed that Canagliflozin could reverse kidney oxidation damage in isoproterenol-induced mice by elevating the concentrations of endogenous glutathione [41]. In addition, the expression of most dipeptides in the kidney was considerably different after EMPA treatment. Particularly, Val-Tyr (downregulation to upregulation, FC = from 0.24 to 6.94), known as Ang-(3–4), is the shortest peptide segment of local angiotensin (Ang) I and Ang II derivatives, which can bind to AT2R to activate Ca^2+^-ATPase in renal tubular epithelial cells via the cAMP–PKA pathway and antagonist Ang II-mediated Na^+^ reabsorption and affects the downstream signal for RAAS activity to promote urinary sodium excretion and vasorelaxant effect, thereby declining blood pressure [42–44]. In this study, Val-Tyr expression increased remarkably after EMPA intervention. According to previous studies [45], EMPA can promote sodium excretion and decrease blood pressure. Moreover, in a previous study, EMPA treatment inhibited RAAS overactivation in Apo^−/−^ mice but did not influence the expression of Ang II [45]. Therefore, we speculate that EMPA serves as an antagonist of RAAS by improving the metabolism of some dipeptides or stimulating their synthesis.

Tryptophan and its metabolites are precursors to various microbial biosynthetic products and metabolites of the host [46]. The following three pathways are currently associated with tryptophan catabolism: (1) serotonin (5-hydroxytryptamine) metabolism, (2) the indole pathway and (3) the kynurenine (KYN) pathway [47]. Approximately 90% of 5-HT in the body is metabolized by gut bacteria, with the most typical bacteria being *Clostridium sporogenes* belonging to the phylum Firmicutes [46]. In addition, *Clostridium sporogenes* can also utilize tryptophan to produce indoleacetic acid (IAA) and indole-3-propionic acid (IPA) via the indole metabolic pathway [46]. A recent study demonstrated that the proportion of gut bacteria belonging to Firmicutes in db/db mice declined after dapagliflozin intervention [48], which partly explains the downregulation of intermediates in both serotonin (5-hydroxy-l-tryptophan) and indole (indole-3-acetaldehyde) pathways after EMPA treatment in this study. High expression of 5-HT and its end product metabolite (5-hydroxyindoleacetic acid) in the serum is strongly associated with the pathogenesis of renal dysfunction in DM [49], whereas 5-HTR antagonists exert renoprotective effects in DKD [50], suggesting that EMPA plays a renoprotective role by regulating the metabolism of gut bacteria. Most of the tryptophan is degraded via the KYN pathway in the cycle.

Tryptophan, which acts on a series of enzymes, eventually produces acetyl-CoA through the KYN pathway, which completely decomposes to CO_2_ and water after entering the TCA cycle (Fig. 7). Some intermediate metabolites are produced with this process, especially quinolinic acid, which mediates most of the de novo synthesis of NAD+. NAD+ in the kidney is majorly derived from de novo synthesis of NAD+ mediated by tryptophan metabolism [51, 52]. However, a previous study found that P5P phosphatase (a cofactor for KYNase and a key enzyme regulating the 3-hydroxykynurenine metabolism downstream) is inhibited owing to chronic inflammation in DKD, which blocks tryptophan metabolism to produce NAD+ through the KYN pathway [53]. This finding is consistent with the accumulation of KYN and its metabolites (3-hydroxykynurenine [3-HKYN] and kynurenic acid [KYNA]) observed in chronic kidney disease such as DKD [47]. The accumulation of KYN and its metabolites mediate and enhance oxidative stress, immune activation and inflammatory reaction to exacerbate renal damage [47]. In this study, the expression of acetyl CoA (end product of the KYN pathway) and nicotinic acid mononucleotide (NAMN) (downstream metabolites of quinolinic acid) was upregulated in the kidney of db/db mice after EMPA intervention, indicating that EMPA may reverse renal dysfunction by promoting KYN metabolism to ameliorate NAD+ levels.

NAD+/NADH imbalance in DKD manifests as NAD+ decline and NADH overload, which aggravates oxidative stress mediated by ROC in the kidneys and causes mitochondrial dysfunction, energy metabolic disorders and inactivation of various NAD+-dependent enzymes [51]. In a study, impaired NAD+ synthesis in mice with CKD was associated with decreased expression of key enzymes, including quinolinate phosphoribosyl transferase (QPRT) in the NAD+ de novo synthesis pathway and nicotinamide nucleotide adenylyl transferase 1 (NMNAT1) and NMNAT 3 in the salvage synthesis pathway [54]. In this study, the expression of NAMN (precursor to the NAD+ de novo synthesis pathway) and niacinamide (NAM, precursor to the salvage synthesis pathway) was increased after EMPA treatment, indicating that these key enzymes may act as targets for EMPA to amend energy metabolism. In addition, most NAM is mainly produced by NAD+-consuming enzymes, including the superfamily members of sirtuins (SIRT), poly (ADP-ribose) polymerase (PARP) and CD38 [55]. However, in a study, canagliflozin treatment reversed the reduction in SIRT1 expression in db/db mice and improved local NAD+ metabolism in the kidney [56]. We believe this study will provide novel insights into the renoprotection mechanisms of SGLT2 inhibitors.

As shown in Fig. 7, we discovered a dominant accumulation of metabolites after purine degradation in db/db mice, such as xanthine, hypoxanthine and uric acid. However, metabolites involved in ATP degradation-mediated purine nucleotide conversion pathway, including adenosine diphosphate (ADP), adenosine monophosphate (AMP), adenosine (ADO) and adenine (AD), were downregulated after EMPA treatment. EMPA blocks the catabolism of hypoxanthine but improves the recycling of hypoxanthine, which is consistent with the amelioration of ischemia–reperfusion renal injury in mice after intervention with XOR inhibitors [57]. Previous studies have confirmed that the uric acid-decreasing effect of SGLT2 inhibitors is associated with its promoting effects on uric acid excretion and reabsorption inhibition [58, 59]. Based on the data of this study, we speculate that EMPA may reduce uric acid by blocking purine degradation (similar to XOR inhibitors).

Inhibition of the uric acid generation pathway promotes the increase of ATP degradation-mediated purine nucleotide conversion pathway. In the kidneys, the local paracrine release of purine nucleotides is regulated by connexins [60]. Abnormal expression of connexins and the subsequent interruption of intercellular communication in DKD result in the up-regulated expression and activity of hemichannels, which mediate the imbalances for the release of ATP and ADO and may have contributed in part to the pathology of DKD [61–63]. ATP and its degradation metabolite ADO are effective extracellular signaling molecules for purinergic signaling in the kidney, which activate P2 and P1 receptors, respectively, to perform contradictory physiological functions, such as the pro-inflammatory response for ATP and the anti-inflammatory response for ADO [32]. Downregulation of ADO and its receptor A1R is involved in ultrafiltration in the early stages of DKD [64]. A2AR (an adenosine receptor) agonists can attenuate proteinuria and reduce the number of pro-inflammatory cytokines in DKD [65]. Moreover, ADO can be produced by NAD+ metabolism, and enhancing NAD+ metabolism-mediated ADO production has been shown to prevent ischemia-induced acute kidney injury [66]. Researchers have demonstrated that blocking CX43 and its mediated local ATP release from renal tubular epithelial cells can ameliorate renal fibrosis [67]. Interestingly, a recent study observed that EMPA treatment repaired ventricular myocytes' gap junctional intercellular communication and attenuated ventricular fibrosis in mice with metabolic syndrome [68]. Unfortunately, no studies have explored the effects of SGLT2 inhibitors on renal CX43. However, this is undoubtedly an interesting research direction, as the modulation of the local purinergic system by SGLT2 inhibitors may be optimized by using nanotechnology to embed drugs with Cx43 hemichannel blocking effects into nanomaterials with specific targeting to the renal tubules.

A previous study described kidney-specific metabolic reprogramming associated with mitochondrial dysfunctional in db/db mice, manifesting as a compensatory increase in glycolysis and fatty acid metabolism, which was a response to diminished production of ATP induced by dysfunction of the mitochondrial electron transport chain and uncoupling of oxidative phosphorylation [69]. Dihydroorotate dehydrogenase (DHODH) is the first rate-limiting enzyme of pyrimidine de novo synthesis, catalyzing the dihydroorotate oxidized to orotate and further producing various downstream pyrimidine nucleotides [70]. DHODH delivers electrons to ubiquinone during this process and provides reductive ubiquinone for compounds I and III of the respiratory chain, thus coupling the pyrimidine metabolism with mitochondrial phosphorylation [71]. It has been demonstrated that the knockdown of intracellular DHODH partially inhibits the activity of respiratory chain complex III and increases mitochondrial ROS production [72]. However, DHODH inhibitors decline the level of pyrimidine nucleotide but increase the level of upstream metabolites of dihydroorotate [73]. The results of the abovementioned studies are consistent with those observed in this study (the kidney of db/db mice). Furthermore, we found that dihydroorotate levels were significantly increased, whereas the levels of its downstream metabolites were increased, including orotate (despite P = 0.052, it had an increasing trend, with VIP = 1.28 and FC = 1.45), uridine monophosphate (UMP), uracil dinucleotide (UDP) and cytidine monophosphate (CMP). However, the metabolites uridine triphosphate (UTP) and cytidine triphosphate (CTP) involved in the pyrimidine salvage pathway were not detected. Therefore, we speculate that EMPA improves mitochondrial dysfunction and alleviates the metabolic reprogramming of the kidneys in DKD by promoting DHODH-mediated de novo synthesis of pyrimidine to increase mitochondrial electron transport.

Furthermore, in this study, metabolic alterations were observed less in the serum than in the kidney after EMPA treatment. The potential interfered pathway of serum metabolites was ‘glycine, serine and threonine metabolism’, specifically involving the biological transformation of d-serine and l-serine. d-Serine, the most abundant d-amino acid in mammals, is produced from l-serine mediated by serine racemase. The kidney mostly regulates d-serine levels by excreting it through urine or degrading it via d-amino acid oxidase (DAAO, highly expressed in the kidney). However, the intermediate metabolites during d-serine oxidative decomposition, mainly H_2_O_2_, have strong renal toxicity. Previous studies have demonstrated that d-serine concentration is positively correlated with GFR and can serve as a clinical diagnostic biomarker for CKD [74]. However, Tomonori Kimura et al. discovered that the level of d-serine did not increase with a decline in GFR in a proportion of patients with CKD [75]. In this study, we found that d-serine levels increased in serum but were reduced in the kidney in db/db mice. Moreover, the ratio of l-serine to d-serine did not change before and after EMPA treatment (Fig. 7), which is consistent with the results of the aforementioned study [75], collectively indicating that a compensation mechanism in the kidney maintains a certain ratio of l-serine to d-serine. We also found that EMPA significantly reduced the level of local d-serine in the kidney, indicating that it is associated with improving renal function or alterations in l-serine metabolism.

Our study has some limitations. First, the untargeted metabolomics we used is a relative quantification method of the metabolites and needs to be followed up with target validation. Second, the sample size of our study is small, and more animal or clinical samples can be included in the follow-up and combined with some new histological techniques (e.g., spatial omics techniques) for the in-depth study.

In conclusion, this study demonstrated conspicuous metabolic reprogramming in mice with DKD. EMPA treatment improved kidney function and morphology by regulating metabolic reprogramming, including regulation of renal reductive stress, alleviation of mitochondrial dysfunction and reduction in renal oxidative stress reaction. Therefore, this study provides an essential reference for understanding the mechanism of EMPA in renoprotection.

## Supplementary Information

**Additional file 1: Figure S1.** Quality control of kidney and serum proteomics. **Figure S2.** Quality control of kidney and serum metabolomics. **Figure S3.** The identification of differential expressed metabolites between db/db and Empa group.

**Additional file 2: Table S1.** Information of 6009 quantified proteins in renal proteomics.** Table S2.** Information of 1481 quantified proteins in serum proteomics.** Table S3.** Information of 501 and 91 DEPs identified in db/db and Empa groups in renal proteomics, respectively.** Table S4.** Information of 32 EAPs between Empa and db/db groups in kidney.** Table S5.** Information of 235 and 83 DEPs identified in db/db and Empa groups in serum proteomics, respectively.** Table S6.** Information of 51 EAPs between Empa and db/db groups in serums.** Table S7**. Information of 897 quantified metabolites in renal metabolomics.** Table S8**. Information of 678 quantified metabolites in serum metabolomics.** Table S9**. Information of 264 identified differentially expressed metabolites between db/db and db/m groups in kidney metabolomics.** Table S10.** Information of 174 identified differentially expressed metabolites between db/db and db/m groups in serum metabolomics.** Table S11.** Information of 94 Empa-altered metabolites between Empa and db/db groups in kidney.** Table S12**. Information of 37 Empa-altered metabolites between Empa and db/db groups in serum.

## Data Availability

The data supporting the findings of this study are available in Additional files and from the corresponding author.
