# Supplementary material for: SGLT2 inhibitors improve kidney function and morphology by regulating renal metabolic reprogramming in mice with diabetic kidney disease
Source: J Transl Med. 2022 Sep 14;20:420. doi: 10.1186/s12967-022-03629-8 (PMC9476562; doi:10.1186/s12967-022-03629-8)
Supplement: Supplementary file 1 — Additional file 1: Figure S1. Quality control of kidney and serum proteomics. Figure S2. Quality control of kidney and serum metabolomics. Figure S3. The identification of differential expressed metabolites between db/db and Empa group. [file 12967_2022_3629_MOESM1_ESM.docx]

**SGLT2 inhibitors improve kidney function and morphology by regulating renal metabolic reprogramming in mice with diabetic kidney disease**

^#^Yong-Ping Lu^1,2^, ^#^Ze-Yu Zhang^2^, ^#^Hong-Wei Wu^2,3^, Li-Jing Fang^4^, Bo Hu^2^, Chun Tang^1^, Yi-Qing Zhang^1^, [Lianghong Yin](https://pubmed.ncbi.nlm.nih.gov/?sort=date&term=Yin+L&cauthor_id=33614731)^2^, Dong-E Tang^1^, *Zhi-Hua Zheng^3^, *Ting Zhu^1^, *Yong Dai^3^

^1^ Department of Nephrology, Center of Kidney and Urology, the seventh affiliated Hospital, Sun Yat-sen University, Shenzhen, China

^2^ Department of Nephrology, the First Affiliated Hospital of Jinan University, Guangzhou, China

^3^ Guangdong Provincial Engineering Research Center of Autoimmune Disease Precision Medicine, the Second Clinical Medical College of Jinan University, the First Affiliated Hospital of Southern University of Science and Technology, Shenzhen People’s Hospital, Shenzhen, China

^4^ Department of Nephrology, Guangzhou first people's hospital, Guangzhou, China

^#^ Yong-Ping Lu, Hong-Wei Wu, and Ze-Yu Zhang contributed equally to this work.

**Prof.Dr.Yong Dai**

*The First Affiliated Hospital of Southern University of Science and Technology, the Second Clinical Medical College of Jinan University, Shenzhen People’s Hospital, Shenzhen 518020, China, Tel: +86 0755-22942780, E-mail address: daiyong22@aliyun.com*

**Dr. Ting Zhu**

*Department of Nephrology, Center of Kidney and Urology, the seventh affiliated Hospital, Sun Yat-sen University, Shenzhen, China, E-mail address:* ztzoe_093@163.com

**Prof. Dr. Zhi-Hua Zheng**

Department of nephrology, Center of kidney and urology, the seventh affiliated hospital, Sun Yat-sen university, Shenzhen,China, E-mail address: [zhzhihua@mail.sysu.edu.cn](mailto:zhzhihua@mail.sysu.edu.cn)

**Supplementary Figure**

**Supplementary Figure S1. Quality control of kidney and serum proteomics.**

**Supplementary Figure S2. Quality control of kidney and serum metabolomics.**

**Supplementary Figure S3. The identification of differential expressed metabolites between db/db and Empa group.**


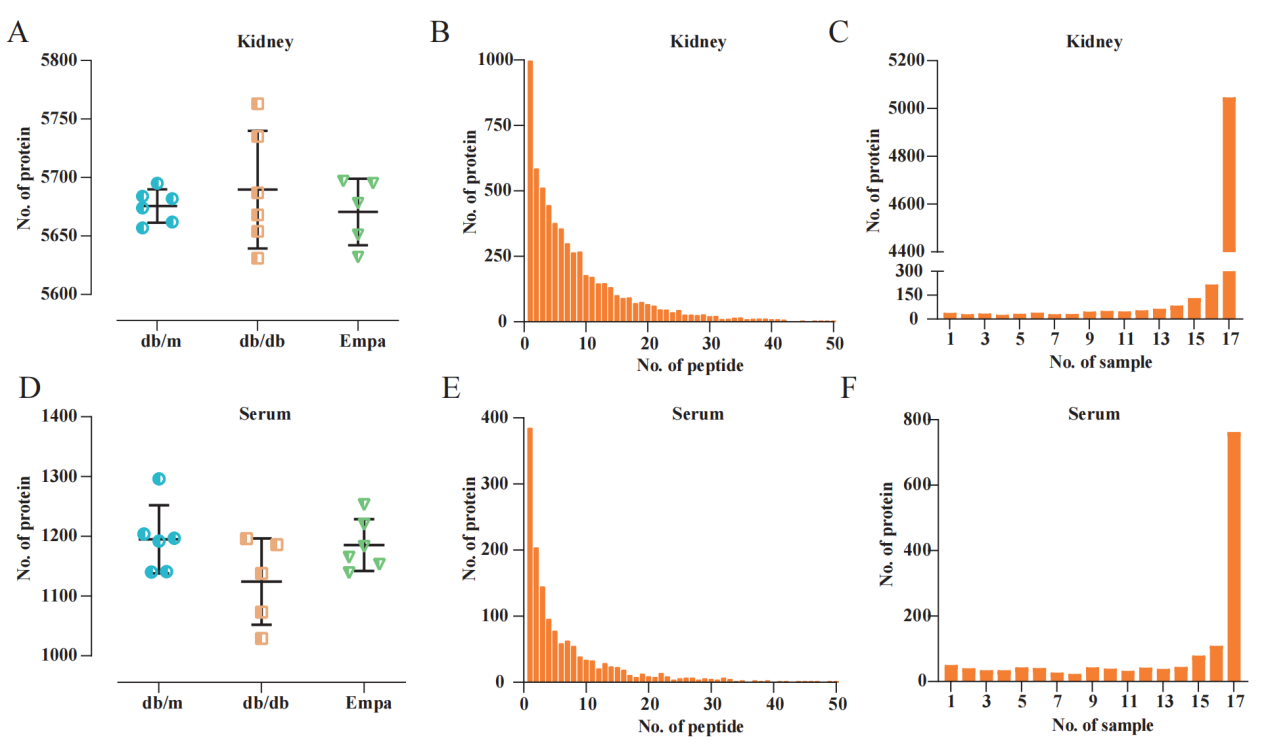


**Supplementary Figure S1: Quality control of kidney and serum proteomics.**

(A and B) The distribution of number of identified proteins in the kidney (A) and serum (B) samples. Each dots with the same color represent multiple independent samples. (C and D) The distribution of peptide numbers of identified proteins in the kidney (C) and serum (D). (E and F) The distribution of protein numbers in the kidney (C) and serum (D) samples.


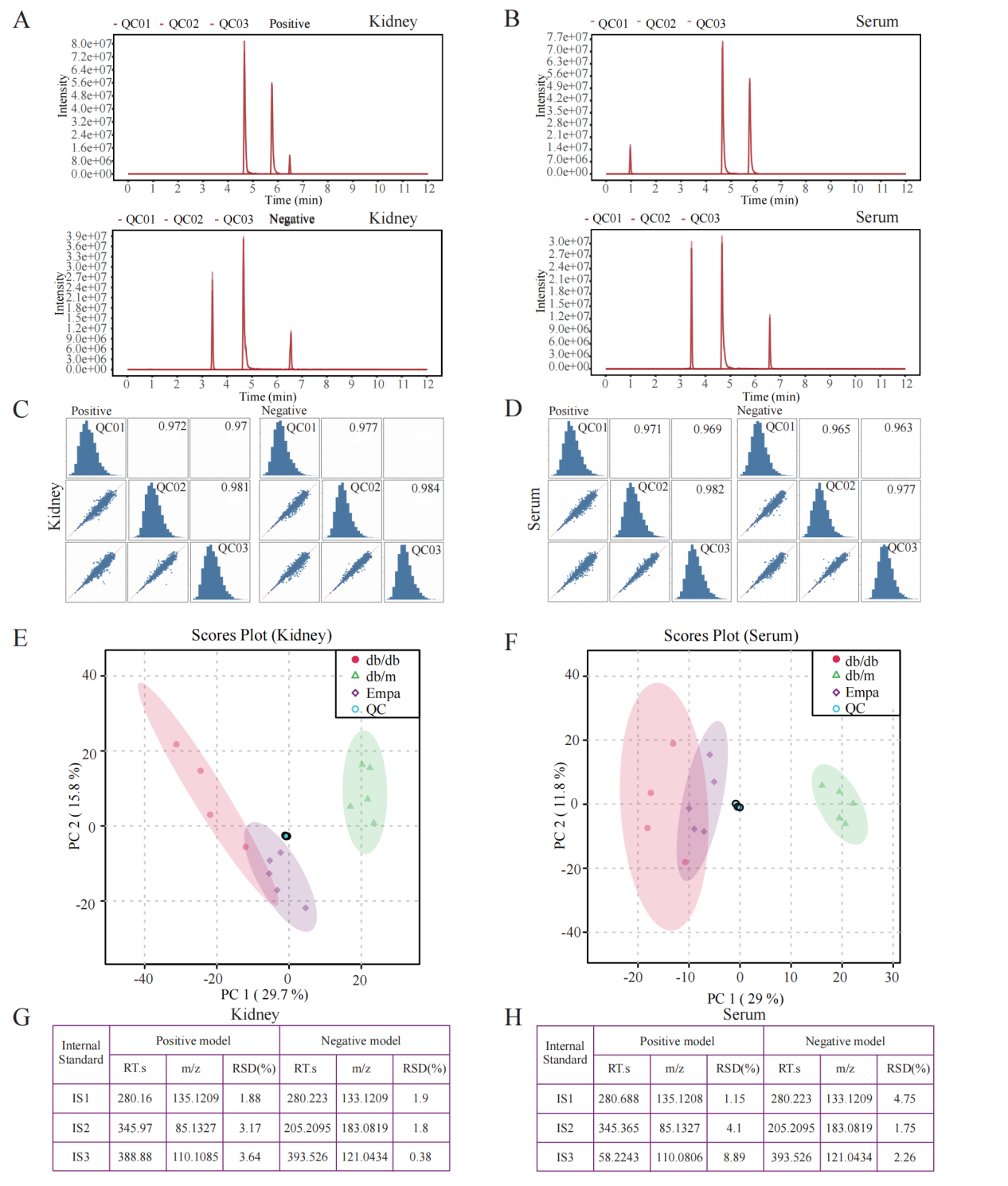


**Supplementary Figure S2: Quality control of kidney and serum metabolomics.**

(A and B) The diagram for the extracted ion chromatography of internal standard metabolites from all quality control samples in the kidney (A) or serum proteomics (B) in both positive ion model and negative ion model. (C and D) The Pearson’s correlation analysis for the QC sample from positive and negative ion models in the kidney (C) and serum (D) metabolomics. (E and F) PCA scores plots showing the metabolic profile for db/m, db/db, Empa, and QC groups in the kidney (E) and the serum (F). All QC samples are tightly clustered in PCA scores plots. (G and H) Response stability of 3 introduced internal standards from positive and negative ion models in the kidney (G) and serum (H) metabolomics. The smaller the relative standard deviation (RSD) among internal standards, the more system stability and higher quality for the data.

**
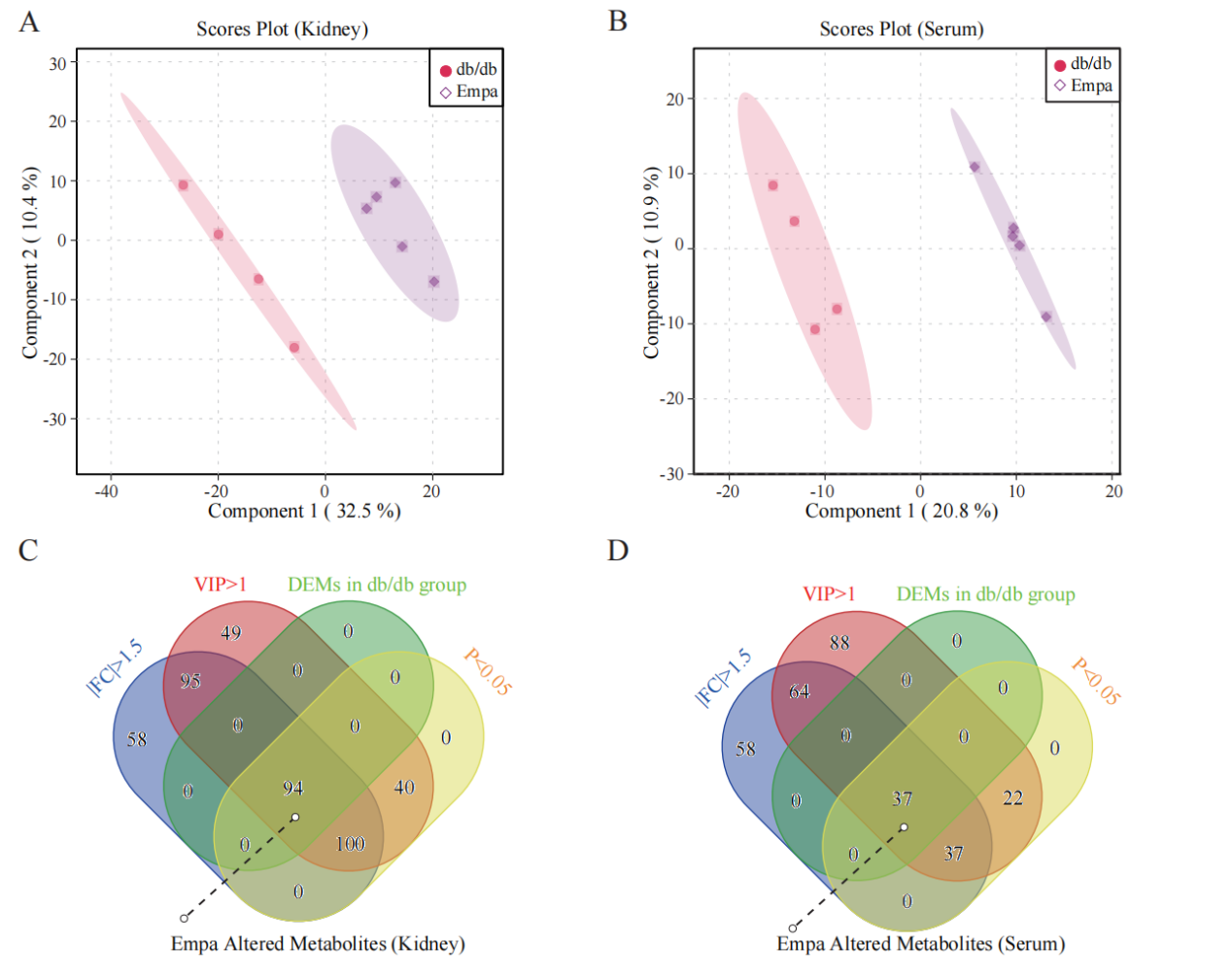
**

**Supplementary Figure S3. The identification of differential expressed metabolites between db/db and Empa group.**

(A, B）PLS-DA score plots for db/db and Empa group in the kidney (A) and the serum (B) metabolomics. (C, D) Venn plot showing the screening methods for 94 and 37 Empa-altered metabolites in the kidney (C) and serum (D), respectively. 194 (kidney) and 74 (serum) differential expressed metabolites between db/db and Empa group was identified with the screening standard of |FC| > 1.5, VIP > 1, *P* < 0.05, and matched in KEGG and HMDB database. Among them, 94 (kidney) and 37 (serum) metabolites, co-expressed in db/db vs. db/m and Empa vs. db/db groups, are considered Empa-altered metabolites.
